# Traumatic brain injury does not disrupt costimulatory blockade-induced immunological tolerance to glial-restricted progenitor allografts

**DOI:** 10.1186/s12974-021-02152-9

**Published:** 2021-04-30

**Authors:** Rui Wang, Chengyan Chu, Zhiliang Wei, Lin Chen, Jiadi Xu, Yajie Liang, Miroslaw Janowski, Robert D. Stevens, Piotr Walczak

**Affiliations:** 1 Russell H. Morgan Department of Radiology and Radiological Science, Johns Hopkins University, Baltimore, MD 21205 USA; 2 Departments of Anesthesiology and Critical Care Medicine, Neurology, Neurosurgery, Johns Hopkins University, Baltimore, MD 21287 USA; 3 Department of Critical Care Medicine, Shengjing Hospital of China Medical University, Shenyang, 110006 Liaoning China; 4 Diagnostic Radiology and Nuclear Medicine, University of Maryland Baltimore, 670 W. Baltimore St., HSF III rm 1176, Baltimore, MD 21201 USA; 5 F. M. Kirby Research Center for Functional Brain Imaging, Kennedy Krieger Institution, Baltimore, MD 21205 USA

**Keywords:** Glial-restricted progenitors, Co-stimulation blockade, Immunological tolerance, Traumatic brain injury, Neuroinflammation

## Abstract

**Background:**

Cell transplantation-based treatments for neurological disease are promising, yet graft rejection remains a major barrier to successful regenerative therapies. Our group and others have shown that long-lasting tolerance of transplanted stem cells can be achieved in the brain with systemic application of monoclonal antibodies blocking co-stimulation signaling. However, it is unknown if subsequent injury and the blood-brain barrier breach could expose the transplanted cells to systemic immune system spurring fulminant rejection and fatal encephalitis. Therefore, we investigated whether delayed traumatic brain injury (TBI) could trigger graft rejection.

**Methods:**

Glial-restricted precursor cells (GRPs) were intracerebroventricularly transplanted in immunocompetent neonatal mice and co-stimulation blockade (CoB) was applied 0, 2, 4, and 6 days post-grafting. Bioluminescence imaging (BLI) was performed to monitor the grafted cell survival. Mice were subjected to TBI 12 weeks post-transplantation. MRI and open-field test were performed to assess the brain damage and behavioral change, respectively. The animals were decapitated at week 16 post-transplantation, and the brains were harvested. The survival and distribution of grafted cells were verified from brain sections. Hematoxylin and eosin staining (HE) was performed to observe TBI-induced brain legion, and neuroinflammation was evaluated immunohistochemically.

**Results:**

BLI showed that grafted GRPs were rejected within 4 weeks after transplantation without CoB, while CoB administration resulted in long-term survival of allografts. BLI signal had a steep rise following TBI and subsequently declined but remained higher than the preinjury level. Open-field test showed TBI-induced anxiety for all animals but neither CoB nor GRP transplantation intensified the symptom. HE and MRI demonstrated a reduction in TBI-induced lesion volume in GRP-transplanted mice compared with non-transplanted mice. Brain sections further validated the survival of grafted GRPs and showed more GRPs surrounding the injured tissue. Furthermore, the brains of post-TBI shiverer mice had increased activation of microglia and astrocytes compared to post-TBI wildtype mice, but infiltration of CD45+ leukocytes remained low.

**Conclusions:**

CoB induces sustained immunological tolerance towards allografted cerebral GRPs which is not disrupted following TBI, and unexpectedly TBI may enhance GRPs engraftment and contribute to post-injury brain tissue repair.

**Supplementary Information:**

The online version contains supplementary material available at 10.1186/s12974-021-02152-9.

## Introduction

The transplantation of stem cells is widely viewed as a promising strategy for treatment of neurological disorders [1]. There has been considerable interest in the precursors of glial cells which might be used to treat a range of hereditary or acquired disorders in which myelination of central nervous system axons is impaired [2]. We have shown that transplantation of human glial-restricted progenitor allografts (GRPs) can significantly ameliorate tissue structure, neurophysiological parameters, and even survival in experimental models of dysmyelination [3] and demyelination [4]. We developed techniques for efficient global delivery of stem cells to the central nervous system [5, 6]. Clinical trials of cell transplantation targeting the brain have suggested a favorable safety profile, but evidence of therapeutic efficacy is limited [1]. A major consideration in achieving therapeutic efficacy is the successful mitigation of allograft rejection [7, 8]. Current clinical immunosuppression protocols have been suboptimal and there is a critical unmet need for new strategies that are pragmatic, effective, and safe.

In recent years, there has been an effort to develop more refined immunomodulation approaches preserving overall functionality of the immune system while inducing selective hyporesponsiveness towards the transplant. These strategies include neonatal desensitization [9], mixed chimerism [10], or interference with co-stimulation signaling [11, 12] with the later shown to be highly effective for protecting transplants. We recently reported successful induction of immunological tolerance towards GRPs using a protocol based on transient blockade of co-stimulation signaling (CD80/CD86 and CD40/CD28) [12]. A remarkable advantage of this protocol is that the co-stimulation blockade (CoB) treatment delivered only at the time of transplantation induces long-lasting tolerance even though CoB inducing molecules are cleared from the body within weeks after administration [13–15]. Of note, grafted cells survived for the duration of the study (200 days) without compromising overall immune defenses and with no toxicity to grafted cells and to the host, bypassing the need for sustained, ongoing treatment that is required in other immune suppression regimens [12]. This novel approach signals an important new direction for allograft cell-based brain regenerative therapy.

While strategies like CoB seem able to induce lasting immunological tolerance, questions have been raised about the robustness of such protocols and whether delayed immune reactivation could occur, leading to potentially catastrophic allograft rejection. For example, established immunotolerance could theoretically be disrupted by pro-inflammatory events unfolding in the brain or systemically, in the setting of traumatic brain injury (TBI). Reactivation of the immune response could be disastrous if large numbers of engrafted cells in the brain were suddenly rejected. The aim of this study was to comprehensively assess the effects of delayed TBI on myelin-deficient mice transplanted with mouse GRPs (mGRPs) and treated with peri-transplantation CoB. We hypothesized that CoB-induced immunological tolerance would be sustained and robust, withstanding exposure to TBI and associated neuroinflammation.

## Materials and methods

**Fig. 1:**
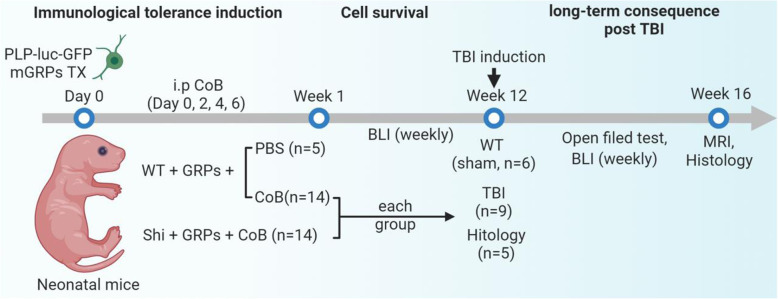
The experimental schematic diagram

Experimental procedures were carried out using our established neonatal transplantation platform with neonatal mice as recipients. MRI and bioluminescent imaging (BLI) were used to non-invasively assess mGRP graft integrity followed by histopathological examination. The experimental design and timeline are schematically represented in Fig. 1.

### Cell culture

Mouse GRPs were isolated from the spinal cord of a proteolipid protein (PLP)­green fluorescent protein (GFP)/β-actin-luciferase transgenic mouse strain at embryonic day 13.5, as previously described [16]. The GFP reporter identifies differentiated oligodendrocytes and luciferase is for detection of engrafted cells via BLI. Cells were cultured in serum-free Dulbecco’s modified Eagle’s-F12 medium (DMEM/F-12) supplemented with N2, B27, bovine serum albumin, heparin, and basic fibroblast growth factor. Immediately prior to transplantation, cells were counted and suspended in phosphate-buffered saline (PBS) at a concentration of 1 × 10^5^ cells/μl. Cell viability was determined by trypan blue exclusion and ensured that more than 95% of them were viable before transplantation.

### Intracerebroventricular cell transplantation

All experiments were performed in accordance with the National Institutes of Health Guide for the Care and Use of Laboratory Animals and approved by the Johns Hopkins Institutional Animal Care and Use Committee. Animals used in this study were male neonatal immunocompetent shiverer (C3Fe.SWV-Mbp^shi^/J) and C57BL/B6J (wildtype, WT). Animals transplanted with mGRPs were distributed in three groups: (1) PBS-treated group (WT + GRPs, *n* = 5), (2) co-stimulation blockade in WT mice group (WT + CoB + GRPs, *n* = 14), and (3) co-stimulation blockade in shiverer mice group (Shi+ GRPs + CoB, *n* = 14). Nine mice in WT + GRPs + CoB and Shi+ CoB + GRPs group were subjected to TBI. Age-matched WT mice (*n* = 6) were used as sham group.

mGRP suspension (4 μl, 10^5^ cells/μl) was stereotaxically transplanted into the newborn pups within 3 days of birth as previously described [3]. Briefly, the pups were cryo-anesthetized and stabilized in a stereotaxic frame. The cell suspension was transplanted bilaterally into lateral ventricles (2 μl/site) using a 31-G Hamilton needle. The co-stimulation blockade agents, anti-mouse-CD154mAb (MR-1, Bio X Cell, 50 μg/mouse) and CTLA4-Ig (ORENCIA, Bristol-Myers Squibb Company, 50 μg/mouse), were administered intraperitoneally at the time of transplantation and on post-operative day 2, 4, and 6, based on our recently published immunological tolerance induction protocol [12]. The pups were returned immediately to their mother after all procedures.

### In vivo bioluminescence imaging (BLI)

Following mGRP transplantation, in vivo BLI was performed weekly to monitor the status of grafted cells. Images were acquired using an IVIS Spectrum/CT instrument (Perkin Elmer). Animals were anesthetized with 2% isoflurane in oxygen then received 150 mg/kg D-luciferin (Gold Biotechnology) injected intraperitoneally. Images were acquired starting 10 min after injection until the peak of the bioluminescence signal occurred. To quantitate the signal of acquired bioluminescence images, regions of interest (ROIs) were drawn over the mouse head and the data were expressed as photon flux (p/s).

### Controlled cortical impact

Traumatic brain injury (TBI) was induced by controlled cortical impact (CCI). Anesthesia was induced with 4% isoflurane and maintained with 1.5–2% isoflurane. The animals were stabilized with ear bars and the skull was exposed by incising the skin along the midline. A 5-mm craniotomy was created midway between the bregma and the lambda on the right side, with the edge of the craniotomy 1 mm lateral to the midline. CCI was carried out using the Benchmark CCI Stereotaxic Impactor (Benchmark Deluxe; My Neurolab, St. Louis, MO, USA). The impact tip (3 mm in diameter) was perpendicularly centered over the exposed brain surface and the injury was produced with the following parameters: 5 m/s velocity, 0.1 s duration, and 2.5 mm depth. Following CCI and the cessation of cortical bleeding, the scalp was sutured. Rectal temperature was monitored and maintained at 37.0 ± 2 °C by an electronic thermostat-controlled heating pad (Stoelting Co., Wood Dale, IL, USA) throughout the experimental and recovery periods. Sham-operated animals underwent the identical anesthetic and surgical procedures without CCI.

### Open-field test

On the day prior to CCI or sham procedures, each mouse was placed in an open-field (OF) environment for acclimation and baseline measurements. Subsequent tests were performed on days 7, 21, and 28 following CCI or sham procedures. The mice were individually placed into the center of the apparatus (40 cm × 40 cm) to explore for 10 min. The movements of the animals were recorded by an overhead video camcorder. The traveling time in the software-defined 20 × 20-cm center region of the apparatus was recorded for each animal and the time spent in the center region was measured. Normalized data (relative to the baseline) were used for statistical analysis.

### Magnetic resonance image acquisition

In vivo MRI was performed on a horizontal a Bruker 11.7-T MRI scanner with a 23-mm mouse brain surface transceiver coil. During imaging, mice were anesthetized with 2% isoflurane and positioned in an animal holder with circulating warm water (Bruker Biospin). T2 (TR/TE = 2500/30 ms, field of view (FOV) = 14 × 14 mm^2^, matrix = 256 × 256) and T1 (TR/TE 350/6.7 ms, FOV = 14 × 14 mm^2^, matrix = 128 × 128)-weighted images of brain were acquired. To visualize BBB status, mice received 0.07 ml of gadolinium (Gadoteridol, Bracco) intraperitoneally and T1-weighted images were repeated 10 min post-gadolinium as previously reported [17, 18]. All the images were processed using Image J software (https://imagej.nih.gov/ij/).

#### Brain histology and immunofluorescence

Animals were anesthetized and perfused transcardially with 5% sucrose followed by 4% paraformaldehyde (PFA). The brains were rapidly dissected and post-fixed overnight in 4% PFA at 4 °C. The brains were cryopreserved in 30% sucrose and then cut into 30-μm thick coronal sections. Hematoxylin and eosin (HE) staining was performed according to standard protocols to assess the volume of damage post-CCI. The stained sections were observed and acquired under light microscopy. For immunofluorescence staining, sections were first blocked with 5% bovine serum albumin (BSA) in 0.1% Triton X-100 for 1 h. The sections were then incubated over night at 4 °C with primary antibodies, rabbit anti-GFAP (1:250, Dako), rabbit anti-IBA1 (1:250, Wako), and rabbit anti-CD45 (1:150, Abcam) to mark astrocytes and microglia, respectively. Then, sections were incubated for 2 h with Alexa-594 (1:250, Invitrogen) secondary antibodies at room temperature. Immunofluorescence images were acquired using a Zeiss Apotome 2 fluorescent microscope. Fluorescent images were analyzed with Image J for quantification of fluorescent intensity and cell counting.

### Statistical analysis

Data are presented as mean ± standard deviation and were statistically analyzed using GraphPad Prism 8.0 (GraphPad Software, Inc., La Jolla, CA, USA). For comparison between two groups, a two-tailed Student’s *t*-test was performed. For multiple comparisons, one-way ANOVA was used followed by Tukey’s post hoc test. A value of *p* < 0.05 was considered statistically significant.

## Results

### Survival of transplanted mGRPs pre- and post-TBI

**Fig. 2:**
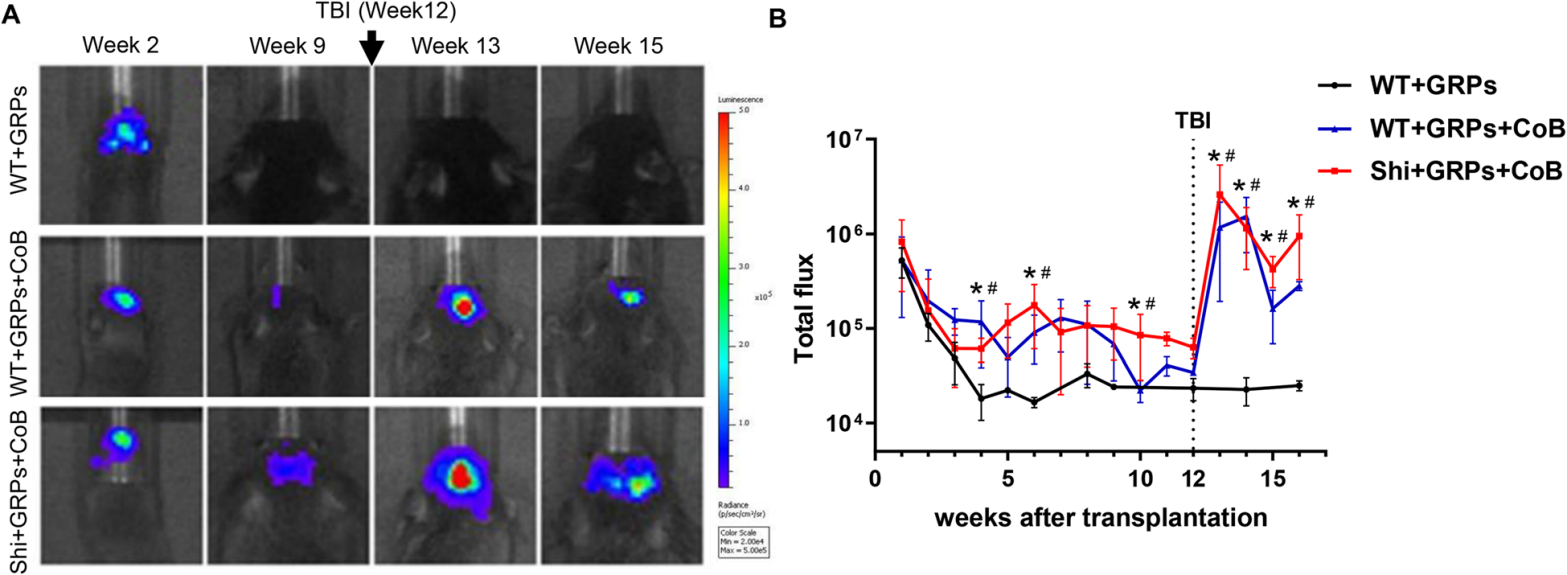
Survival of transplanted mGRPs monitored by BLI. **a** Representative BLI scans of mice transplanted with GRPs at indicated time points. **b** Quantification of BLI signal for each group. Statistically significant difference in WT + GRPs + CoB (*p < 0.05, *n* = 5) and Shi + GRPs + CoB (^#^*p* < 0.05, *n* = 5) compared with WT + GRPs

In mGRP-grafted immunocompetent C57BL/6J (WT, *n* = 5) mice, which did not receive CoB, BLI demonstrated a gradually declining signal which dropped to baseline at week 4 (Fig. 2a, b), indicating complete graft rejection. By contrast, in the WT + GRPs + CoB (*n* = 5) and Shi + GRPs + CoB (*n* = 5) groups, the BLI signal remained constant and above the background until 12 weeks post-grafting (Fig. 2a, b). Both CoB-treated groups showed a statistically significant difference from the WT group without CoB (*p* < 0.05) at the indicated timepoints (Fig. 2b). The data suggested that CoB-induced immunotolerance resulted in the survival of allograft mGRPs.

Twelve weeks after neonatal transplantation with allogeneic mGRPs, mice were subjected to the TBI procedure. The BLI signal (in photon flux) before TBI at week 12 was 6.29e10^4^ ± 6.9e10^3^ in Shi + GRPs + CoB group and 1.98e10^4^ ± 2.82e10^3^ in WT + CoB group. Surprisingly, a significant increase in BLI signal was observed 1 week after TBI (3.13e10^6^ ± 1.4e10^6^ and 2.40e10^4^ ± 3.8e10^3^, respectively). The BLI signal subsequently decreased but still remained higher than the preinjury level (Fig. 2a, b). These results suggested that following TBI, the engrafted mGRPs were not rejected.

### Behavioral assessment

**Fig. 3:**
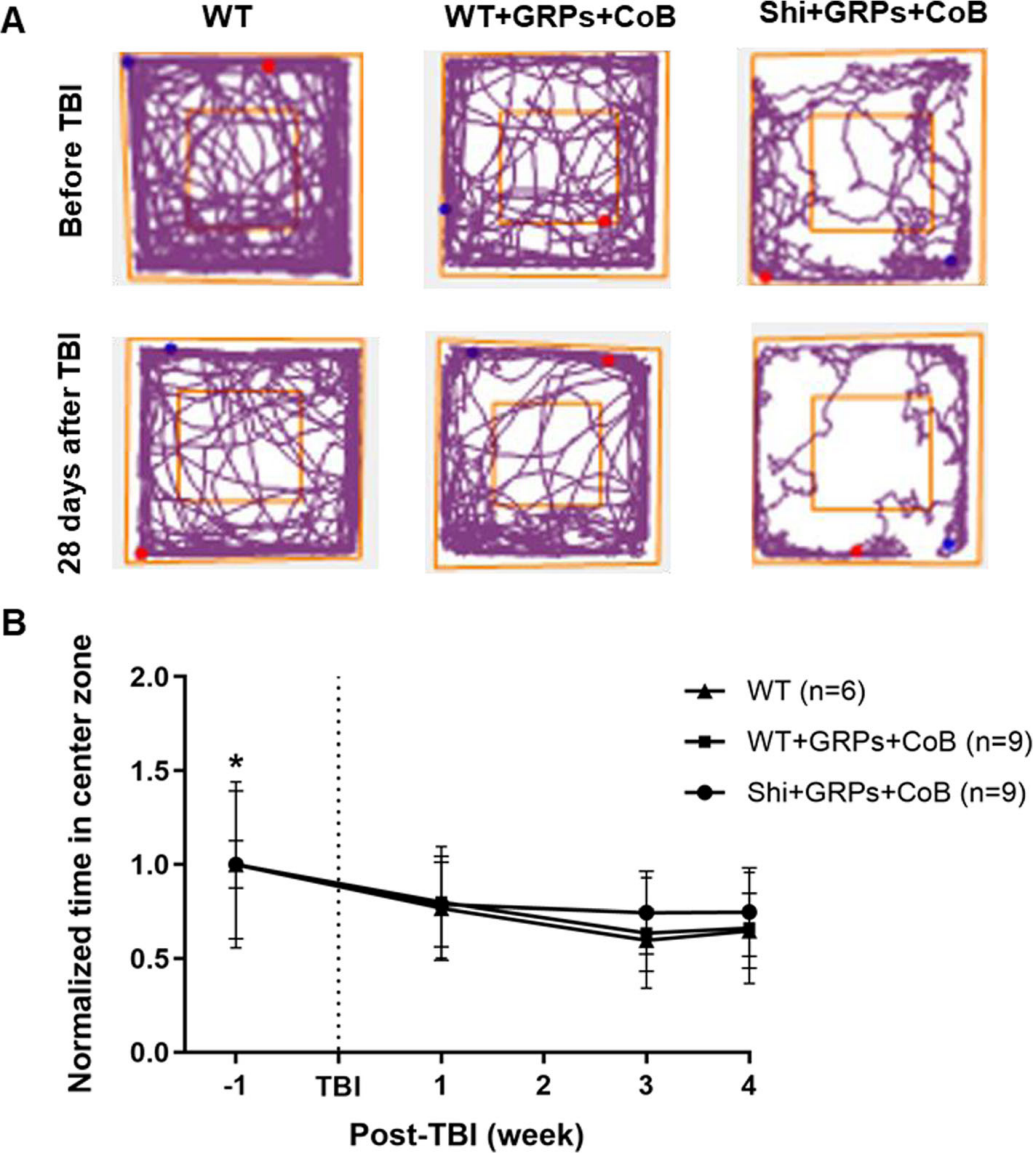
TBI induces anxiety-like behavior in open-field test. **a** Representative trace plots from one mouse in each group during performance of an open-field test. **b** The time spent in the center during tests at indicated week (weeks − 1 and 0 were designated as the day prior to TBI and the date of TBI, respectively) was normalized to the baseline (prior to TBI). *p < 0.05 compared with the different points post-TBI in each group

To evaluate anxiety-like behavior, open-field test was performed prior to TBI surgery and on week 1, 3, and 4 after TBI surgery. The time spent in the central region of the testing chamber for each animal was measured and represented as the ratio relative to the baseline. The travel pathway for all mice groups showed reduced exploratory activity in the center after TBI, indicating anxiety-like behavior (Fig. 3a). A significant decrease was determined in each group post-TBI compared with prior to TBI (Fig. 3b, *p* < 0.05). Importantly, there was no statistically significant difference between each group at all indicated timepoints after TBI irrespective of GRP transplantation or CoB administration (Fig. 3b, *p* > 0.05), suggesting a lack of detrimental behavioral effects of cell engraftment or CoB.

### MRI and histopathological assessment of TBI-induced lesion

**Fig. 4:**
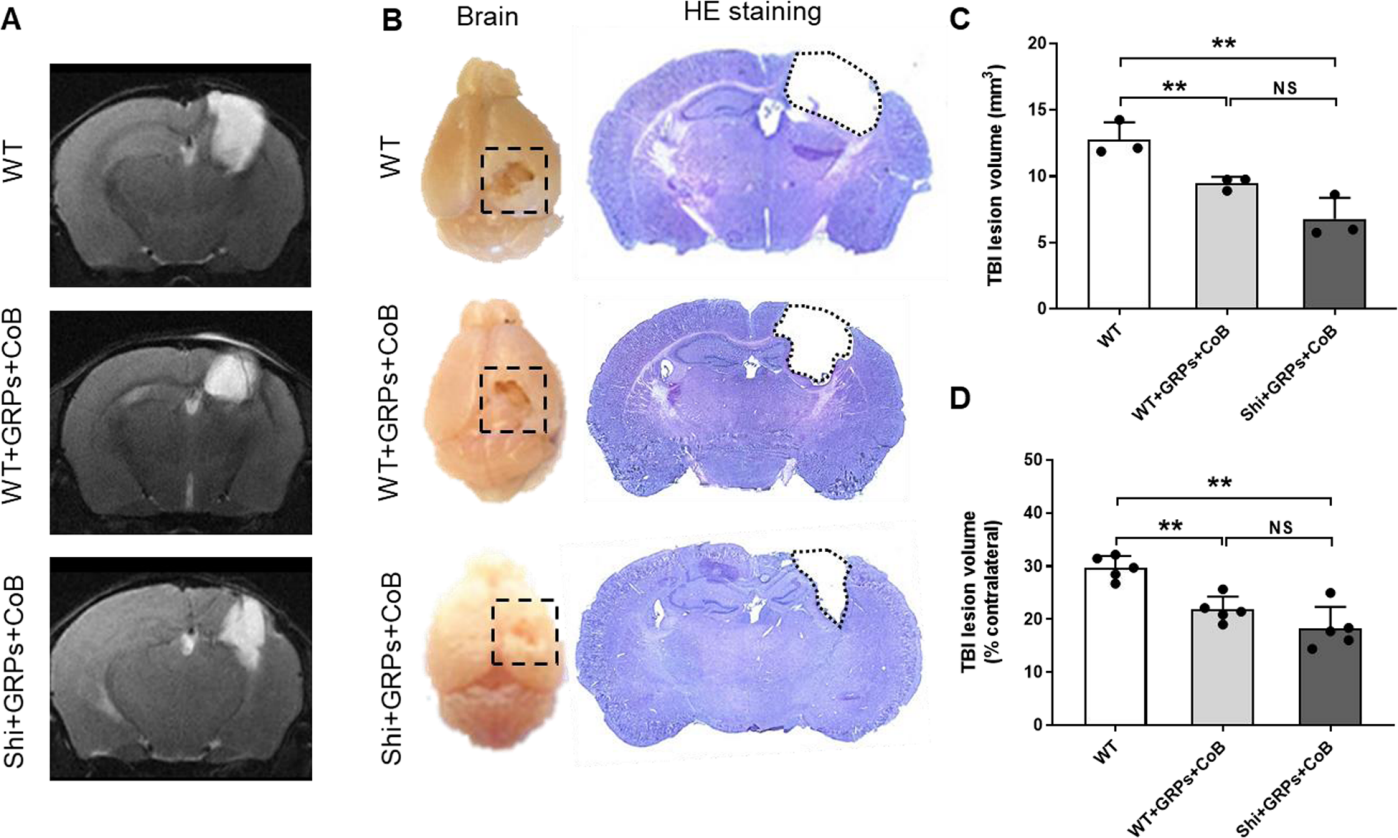
MRI and HE staining for TBI lesion assessment. Representative MR images **a** and HE staining **b** of mice subjected to TBI. Quantification of TBI lesion based on MRI (**c**, *n* = 3/group) and HE staining (**d**, *n* = 5/group). NS, no significant difference; **p < 0.01

MRI and histological analysis were used to investigate the effect of mGRP transplantation and CoB on TBI-induced tissue damage. As expected, the CCI procedure resulted in a visible brain cavity 28 days later in all groups (Fig. 4a, b). Notable on T2-weighted MRI (Fig. 4a) and HE-stained coronal brain sections (Fig. 4b) was the smaller damage in transplanted mice, as evidenced by a decrease in the lesion volume in GRP-transplanted mice in contrast to the non-transplanted WT mice (*p* < 0.05, Fig. 4c, d). Additionally, contrast (gadolinium)-enhanced MRI showed only mild marginal enhancement at the border of the lesion in all injury groups (Suppl. Fig. 1), implying that the blood-brain barrier was not significantly breached. These data suggest that the engrafted GRPs did not exacerbate TBI-induced brain damage and may in fact contribute to post-TBI tissue repair.

### Histological validation of GRPs survival and distribution

**Fig. 5:**
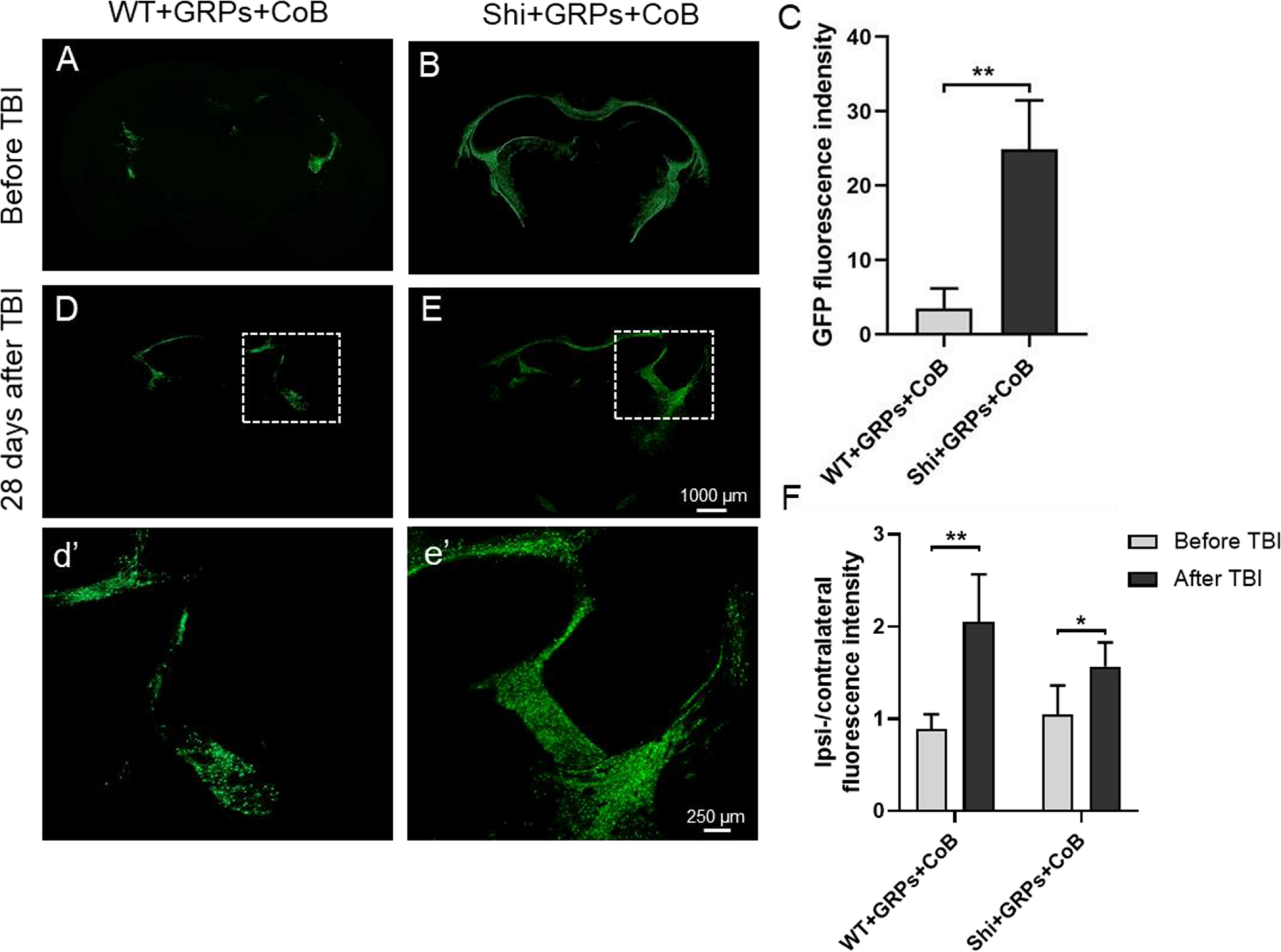
Immunofluorescence for grafted PLP-GFP cells before and after TBI. **A**, **B** Brain sections from the WT + GRPs + CoB and Shi + GRPs + CoB group at 12 weeks of age (before TBI). **C** Quantitative analysis of the mean intensity of GFP fluorescence in different groups. **D**, **E** 28 days after TBI, brain sections exhibited the distribution of grafted cells (PLP-GFP, green). **d**’, **e**’ Insets from **D** and **E**, respectively. **F** Quantitation of ipsi/contralateral fluorescence intensity in each group before and after TBI. *n* = 5/group, *p < 0.05, **p < 0.01

Before TBI, cells were visible in WT + GRPs + CoB group and the grafted GRPs (PLP-GFP, green) in Shi + GRPs + CoB group survived and colonized periventricular structures of the brain including corpus callosum, internal capsule, and fimbria (Fig. 5A, B). The engraftment and maturation of transplanted GRPs in WT + GRPs + CoB group were less pronounced compared to that of Shi + GRPs + CoB group (Fig. 5A, B), as evidenced by lower GFP fluorescence intensity driven by oligodendrocyte-specific PLP promoter (Fig. 5C, *p* < 0.05). Four weeks after TBI, the grafted cells were not rejected (Fig. 5D, E). Importantly, histology showed more GRPs surrounding the injured tissue (Fig. 5D, E), as reflected by an increase in the ipsi-/contralateral GFP fluorescence intensity after TBI (Fig. 5F). The findings further support the possibility of GRPs facilitating tissue repair, consistent with the decreased lesion volume in GRP-transplanted group.

### Neuroinflammation assessment in transplanted mice subjected to CoB after TBI

**Fig. 6:**
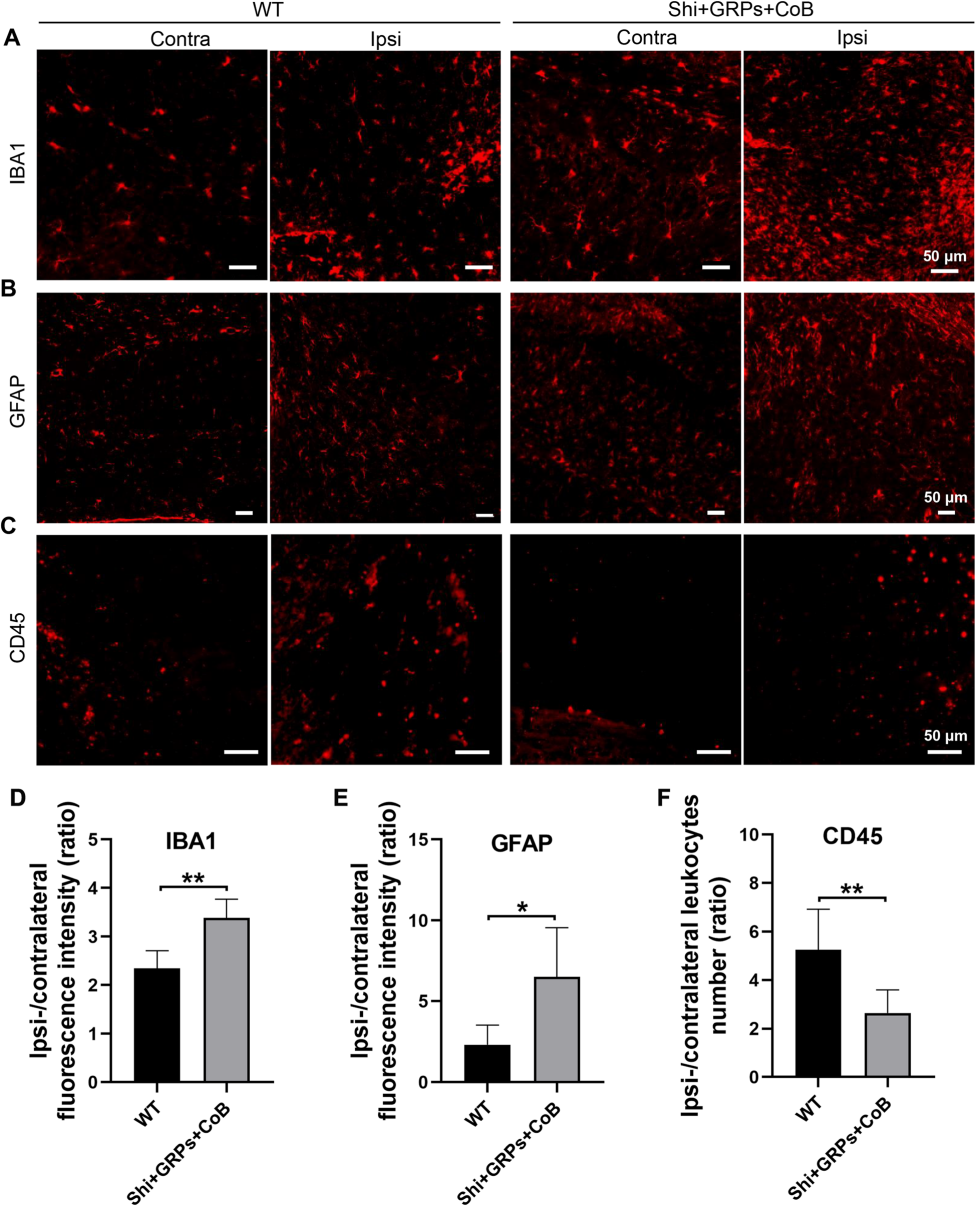
Histological assessment of neuroinflammation. **a**–**c** Representative images of IBA1, GFAP, and CD45 fluorescent staining for neuroinflammation. **d**–**f** Quantitative analysis of the ipsi-/contralateral IBA1, GFAP fluorescence intensity and CD45^+^ cell number between the WT and at 28 days post-TBI. *n* = 6/group, *p < 0.05, **p < 0.01

To observe the local immune status in response to TBI, neuroinflammation was assessed by histological staining of WT and Shi + GRPs + CoB mice 28 days after TBI. In both groups increased levels of activated microglia (IBA1^+^), astrocytes (GFAP^+^), and leukocytes (CD45^+^) were noted in the area close to the TBI lesion relative to the corresponding area in the healthy hemisphere (Fig. 6a–c), as evidenced by a fluorescence intensity or cell number (Suppl. Fig. 2). The results indicate a persistent chronic inflammation after TBI. Interestingly, we found a pronounced ratio of ipsi-/contralateral fluorescence intensity for IBA1 and GFAP, but a lower ratio for CD45 in Shi + GRPs + CoB vs. WT (Fig. 6d–f).

## Discussion

These findings demonstrate that CoB induces sustained immunological tolerance to allogeneic GRPs transplanted into the brain of immunocompetent mice, and that this tolerance was maintained in the setting of delayed TBI associated with a profound neuroinflammatory response. These results support our hypothesis that long-term and robust immunological tolerance to allograft can be achieved by CoB.

The transplantation of allogeneic stem cells is a highly promising strategy for the treatment of neurological disorders of the brain. A significant body of research indicates that grafted cells can effectively promote structural and functional recovery in injured host brains [1]. However, the efficacy of cell-based therapy is highly dependent on effective and safe mitigation of graft rejection. Conventional immunosuppression approaches have had inconsistent results in facilitating cerebral graft survival and produce many side effects. Our group previously developed a strategy using a brief course of peri-transplantation CoB (CTLA4-Ig and MR-1), successfully preventing the rejection of GRPs transplanted in adult dysmyelinated mice brains [12]. The intracerebral GRPs survived for more than 200 days and initiated widespread myelination by differentiating into mature oligodendrocytes [12]. While long-term graft survival was achieved, a critical question is whether the established immunological tolerance might be compromised or disrupted in the presence of a significant pro-inflammatory event. The abrupt loss of immunological tolerance to cells transplanted in the brain might have dramatic and disabling neurological and behavioral effects and could be difficult to mitigate.

In the present study, neonatal mice transplanted with mGRP and receiving CoB had histological and BLI evidence of successful engraftment at 12 weeks. Histology demonstrated that the transplanted GRPs successfully engrafted the host brain and migrated throughout the corpus callosum. Furthermore, we found a higher number of GFP^+^ GRPs in the shiverer mice than that in the WT mice. Since the expression of GFP was under the oligodendrocyte-specific PLP promoter, this result suggested that the microenvironment in the dysmyelinated mice brains might preferentially drive GRP differentiation towards the myelinating oligodendrocyte phenotype.

It is well known that TBI can trigger significant neuroinflammation by activating innate and adaptive immunity with concomitant infiltration of immune cells and heightened inflammatory signaling [19]. We induced TBI with the goal of exploring the effects on GRP engraftment of a local immune response in the brain. The increased signal detected on BLI scans and histopathological analysis indicated TBI-associated neuroinflammation (elevated microglial and astrocytic markers in the ipsilateral hemisphere), but the latter did not trigger rejection of the grafted cells and may even have induced a GRP proliferative response. The steep rise in the BLI signal 1 week post-TBI might reflect other mechanisms including reduced light absorption due to the craniectomy and scattering, and an increase in GRP metabolic state. Yet studies have shown that in response to TBI, quiescent stem and progenitor cells may be activated and divide [20, 21]. Hence, post-TBI expansion of transplanted GRP populations seems at least plausible and warrants further investigation in this context.

The open-field test was performed in our study to measure anxiety-like behavior. Consistent with previous studies [22, 23], we found that TBI resulted in enhanced anxiety-like behavior. It is noteworthy that neither GRP transplantation nor CoB altered mice open-field behavior as a comparable assessment index (time spent in center zone) in any groups. These findings illustrate that engrafted cells and CoB did not contribute to behavioral changes after TBI. While more subtle neurological function assessment tests are available, here our primary focus on identifying potential overall detrimental effects associated with graft rejection rather than amelioration of traumatic brain injury.

MRI and histological results suggested that GRP transplantation may have contributed to an alleviation of TBI-induced brain damage, as reduced lesion volume was demonstrated. Histology at 4 weeks after TBI demonstrated survival of grafted GRPs, corroborating the BLI result. Interestingly, in comparison to a symmetrical distribution of GRPs in the brain prior to TBI, following TBI a higher number of GRPs converged in peri-lesioned tissue in the ipsilateral hemisphere. These observations support the notion that grafted cells might contribute to tissue repair by proliferating and migrating to the site of injury; they are consistent with the reduction in TBI lesion volume observed in GRP transplantation group. GRP-associated post-TBI repair may reflect a process of remyelination which we have demonstrated in other studies [3, 12] and could represent a therapeutic advance for TBI which is characterized by widespread demyelination [24, 25]. These postulated therapeutic effects of GRPs may compound neurotrophic and supportive roles in neuroprotection, as reported elsewhere [3, 4, 26].

Our study further explored the effect of grafted GRPs combined with CoB on neuroinflammation after TBI. In agreement with prior studies [27], local neuroinflammation involving the activation and recruitment of resident glia (microglia and astrocytes) and infiltration of leukocytes was observed at 4 weeks post-TBI. We also found that glial infiltration was more intense in the transplanted shiverer group with CoB than WT group, as has been reported elsewhere [28]. In contrast to glial activation, infiltration of CD45^+^ leukocytes was less pronounced in the transplanted shiverer group with CoB in comparison to WT group, consistent with our prior work [12].

## Conclusions

This study showed that immunological tolerance of brain-allografted GRPs induced by CoB was not disrupted following TBI. Our results also suggested unexpectedly that TBI may enhance GRPs engraftment and contribute to post-injury brain tissue repair.

## Supplementary Information

**Additional file 1: Figure S1.** Coronal view of T1-weighted brain MRI with and without gadolinium 28 days post TBI showed a lack of blood-brain barrier (BBB) breakdown. **Figure S2.** Quantitative comparison of IBA1 fluorescence intensity (A), GFAP fluorescence intensity (B) and CD45+ cell number (C) between ipsi- and contralateral hemisphere in shiverer (*n* = 6) and wildtype (*n* = 6) groups **P < 0.01.

## Data Availability

The datasets used and/or analyzed during the current study are available from the corresponding author on reasonable request.

## Abbreviations

TBI: Traumatic brain injury
GRPs: Glial-restricted precursor cells
CoB: Co-stimulation blockade
CTLA4-Ig: Cytotoxic T lymphocyte-associated antigen 4-immunoglobulin
CD154mAb: CD154 monoclonal antibody
PBS: Phosphate-buffered saline
WT: Wildtype
Shi: Shiverer
MRI: Magnetic resonance image
BLI: Bioluminescence imaging
CCI: Controlled cortical impact
HE: Hematoxylin and eosin
PFA: Paraformaldehyde
GFP: Green fluorescence protein
GFAP: Glial fibrillary acidic protein
IBA-1: Ionized calcium binding adaptor molecule 1
