# Supplementary material for: Traumatic brain injury does not disrupt costimulatory blockade-induced immunological tolerance to glial-restricted progenitor allografts
Source: J Neuroinflammation. 2021 Apr 30;18:104. doi: 10.1186/s12974-021-02152-9 (PMC8088005; doi:10.1186/s12974-021-02152-9)
Supplement: Supplementary file 1 — Additional file 1: Figure S1. Coronal view of T1-weighted brain MRI with and without gadolinium 28 days post TBI showed a lack of blood-brain barrier (BBB) breakdown. Figure S2. Quantitative comparison of IBA1 fluorescence intensity (A), GFAP fluorescence intensity (B) and CD45+ cell number (C) between ipsi- and contralateral hemisphere in shiverer (n = 6) and wildtype (n = 6) groups **P < 0.01. [file 12974_2021_2152_MOESM1_ESM.docx]

Supplementary Materials

**Traumatic Brain Injury Does Not Disrupt Costimulatory Blockade-Induced Immunological Tolerance to Glial-Restricted Progenitor Allografts**

Rui Wang^1,2,3,5#^, Chengyan Chu^1,2#^, Zhiliang Wei^2,4^, Lin Chen^2,4^, Jiadi Xu^2,4^, Yajie Liang^1^, Miroslaw Janowski^1^, Robert D. Stevens^2,3,4^, Piotr Walczak^1*^

^1^ Diagnostic Radiology and Nuclear Medicine, University of Maryland Baltimore, Baltimore, MD 21201, USA.

^2^ Russell H. Morgan Department of Radiology and Radiological Science, Johns Hopkins University, Baltimore, MD 21205, USA.

^3^ Departments of Anesthesiology and Critical Care Medicine, Neurology and Neurosurgery, Johns Hopkins University School of Medicine, Baltimore, MD 21287, USA.

^4^ F. M. Kirby Research Center for Functional Brain Imaging, Kennedy Krieger Institution, Baltimore, MD 21205, USA.

^5^ Department of Critical Care Medicine, Shengjing Hospital of China Medical University, Shenyang, Liaoning 110006, China.

^#^Rui Wang and Chengyan Chu contributed equally to this work.

**^*^Correspondence to:**

Piotr Walczak, MD, Ph.D.

University of Maryland Baltimore 670 W. Baltimore St., HSF III rm 1176

Baltimore, MD 21201

Phone +1 (410) 706 7904

Email: pwalczak@som.umaryland.edu


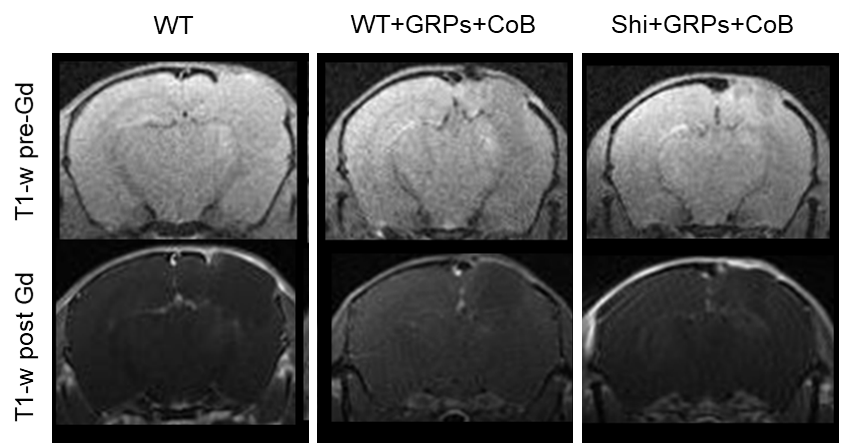


**Figure S1. Coronal view of T1-weighted brain MRI with and without gadolinium 28 days post TBI showed a lack of blood-brain barrier (BBB) breakdown.**


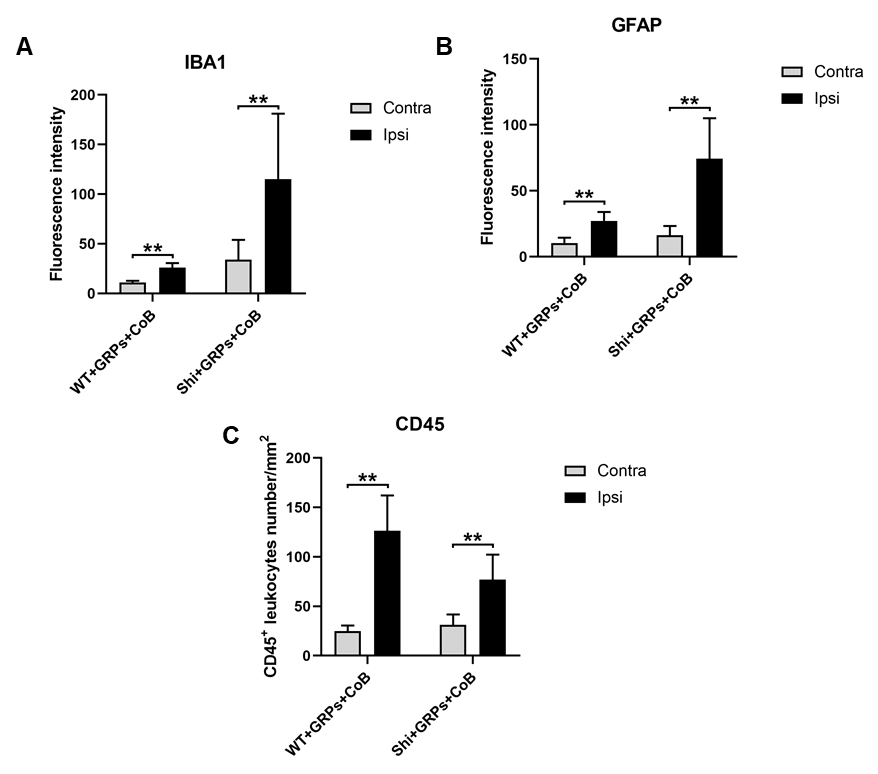


**Figure S2. Quantitative comparison of IBA1 fluorescence intensity (A), GFAP fluorescence intensity (B) and CD45^+^ cell number (C) between ipsi- and contralateral hemisphere in shiverer (n=6) and wildtype (n=6) groups **P < 0.01.**
